# Exploring Serious Games in Supporting Postnatal Depression: Narrative Review

**DOI:** 10.2196/70777

**Published:** 2026-01-16

**Authors:** Weijie Wang, Erika Penney, Valerie Gay, Jaime Garcia

**Affiliations:** 1 School of Computer Science, Faculty of Engineering and IT (Information Technology), University of Technology Sydney, 15 Broadway, Ultimo, Sydney, 2007, Australia, 61 0411278545; 2 Graduate School of Health, Faculty of Health, University of Technology Sydney, Sydney, Australia; 3 School of Electrical and Data Engineering, Faculty of Engineering and IT (Information Technology), University of Technology Sydney, Sydney, Australia

**Keywords:** serious game, postnatal depression, digital health, mobile phone, perinatal education, mental health

## Abstract

**Background:**

Postnatal depression (PND) is a clinical sign of sadness in certain individuals after childbirth. PND affects the mother, the baby, and the whole family. PND is now recognized as a public health concern worldwide. The global prevalence of PND is approximately 17.22%. However, less than half of those affected seek help, which means over 50% of PND cases are left untreated. Current reviews lack focus on digital interventions targeting parents in late pregnancy or postnatal stages. Existing studies prioritize symptom relief over fostering help-seeking behaviors.

**Objective:**

This study aims to identify what serious games have been applied to support the treatment or help-seeking of PND and what gaps are still left.

**Methods:**

Eligibility criteria for this review included full-text papers from 2015 to 2024 from conferences or peer-reviewed journals that were relevant to serious games to support help-seeking behaviors for individuals with depression. Seven research databases and publisher repositories were used. The final search was conducted in March 2025, and a thematic analysis was used to identify and organize recurring themes. As this review adopts a narrative approach, predefined eligibility criteria, a structured search strategy, and review by an interprofessional team were used to reduce selection bias.

**Results:**

Only 2 studies related to PND were identified. After expanding the search string to depression, 13 studies were included in this review, and the studied games were divided into 3 help-seeking categories: promoting knowledge, reducing stigma, and raising awareness. This review identified that gamification, educational messages, and supportive character interactions could enhance engagement, build coping skills, and promote help-seeking in a practical, parent-friendly format. Nonetheless, this paper is limited by the reliance on depression literature due to scarce PND-specific studies, the quality of included studies, the exclusion of non-English language publications, and the use of common but select academic databases. These factors may affect generalizability but also serve to highlight critical gaps for future research and targeted intervention design.

**Conclusions:**

There is a dearth of studies directly related to PND. Existing games commonly use narrative storytelling and interactive scenarios to promote empathy, correct misconceptions, and encourage help-seeking in broad depression. However, few are designed specifically for new parents, whose unique needs—such as time constraints—make mobile platforms the most suitable format for effective engagement. The authors propose that the future interprofessional codevelopment of a mobile serious game tailored to new parents would address the intervention and literature gaps identified in this review. It is argued that key design elements should include an emotionally engaging narrative, meaningful player choices, real-life parenting scenarios, calming visuals, and accessible, low-pressure gameplay. This review contributes to the progression of serious game research, with a focus on addressing the needs of an often underserved and undertreated PND population.

## Introduction

### Postnatal Depression: Definition, Consequences, and Treatment

Postnatal depression (PND) is a clinical diagnosis characterized by sadness after the birth of a child [1]. Most cases of PND start between 4 and 8 weeks after childbirth [2]. However, some research suggests that health care providers should screen for PND for up to 3 years after giving birth. This finding is in light of evidence that PND can develop after the traditional 6-month mark in some cases [3]. PND can result in various adverse maternal outcomes, impacting physical health, psychological well-being, interpersonal relationships, and risky behaviors [4]. Moreover, women who experience PND face an increased likelihood of experiencing clinical depression in the future, not just following repeated pregnancies but also throughout other periods of their lives [5].

PND affects not only the mother but the baby and family as a whole [6]. A mother experiencing PND may have difficulty bonding with her infant, providing proper care such as breastfeeding, and playing other maternal roles [1]. The potential consequences of this situation may extend to the child’s physical, cognitive, language, emotional, social, and behavioral development and even impact the dynamic within the family unit over an extended period of time [7].

Perinatal mental health issues are now recognized as a significant public health concern all over the world. The global prevalence of PND was found to be approximately 17.22% in the largest meta-analysis of PND to date [8]. It is estimated that perinatal mental health disorders cost the Australian economy Aus $877 million per year (a currency exchange rate of Aus $1=US $0.66 was applicable) [9], the United States economy US $14.20 billion per year [10], and the United Kingdom £8.10 billion each year (a currency exchange rate of UK £1=US $1.34 was applicable) [11].

### Health-Seeking Behaviors and PND

However, less than half of those affected seek help, which means over 50% of PND is left untreated [12]. The barriers to help-seeking for PND are various, including social and cultural stigma, lack of knowledge and awareness, and lack of accessibility to the health care system [13]. As highlighted, PND can have a detrimental effect on the entire family. Thus, further research is needed on the most effective ways to empower expectant new parents to seek help earlier and maintain positive mental health, ensuring this condition does not remain a silent struggle.

### Digital Interventions and Management of PND

Digital games are interactive, computer-based experiences in which players interact with a virtual environment through input devices such as keyboards, controllers, or touchscreens. They range from simple, text-based applications to complex, immersive worlds powered by advanced graphics and artificial intelligence. Digital games can be played on multiple platforms, including personal computers, gaming consoles, smartphones, and virtual reality (VR) systems.

Game development is a complex process that often leverages specialized tools to streamline workflows and enhance functionality. Two critical components in this ecosystem are middleware solutions and game engines. Middleware provides developers with prebuilt modules to handle specific functionalities, while game engines offer comprehensive frameworks for building games.

Serious games play a crucial role in various domains by blending entertainment with purposeful outcomes, making them valuable tools for education, training, health care, and social awareness [14]. Unlike traditional video games designed solely for recreation, serious games leverage interactive and immersive elements to enhance learning, skill development, and behavioral change. Engaging users in simulated environments provides a risk-free space for experimentation and decision-making, leading to deeper comprehension and improved information retention. This kind of approach makes them particularly effective in fields such as education, where gamified learning experiences can make complex subjects more accessible and engaging for students. In professional training, serious games are widely adopted for skill development and simulation-based learning [15]. Industries such as health care, aviation, and military training use serious games to replicate real-world scenarios, allowing trainees to practice in a controlled setting. For example, medical simulation games help doctors and nurses refine surgical techniques and patient care by providing a safe space rather than performing procedures on real patients [16]. Similarly, corporate training programs integrate serious games to make the traditional learning experience more engaging and dynamic, thereby improving employee knowledge retention and job performance [17].

Beyond education and training, serious games significantly impact mental health and social awareness. Games designed to address depression can provide therapeutic benefits and be valuable and effective in reducing symptoms of depressive disorders [18,19]. Additionally, serious games are used to promote awareness about global challenges, such as climate change and poverty, by immersing players in experiences that foster empathy and drive real-world action [20]. By engaging storytelling, decision-making challenges, and interactive problem-solving, serious games go beyond traditional education and advocacy methods, making complex issues more relatable and actionable for diverse audiences.

In conclusion, serious games are an essential innovation in digital technology, bridging the gap between entertainment and meaningful engagement. Their ability to educate, train, and raise awareness while keeping users involved makes them a powerful tool across various industries. As technology continues to evolve, the potential for serious games to drive positive change in education, health care, and social development will only continue to grow. This narrative review aims to provide a comprehensive and interpretative narrative synthesis of the existing literature on serious games and digital interventions for PND. The goal is to critically evaluate current trends and gaps to explain the need for targeted research in this essential but often overlooked area.

### Knowledge Gap and Objective

There is literature from the past 10 years reviewing digital interventions for symptoms of PND. In 2018, van den Heuvel et al [21] published a systematic review of the current literature on eHealth developments in pregnancy to assess this new generation of perinatal care. Studies that reported using eHealth during prenatal, perinatal, and postnatal timeframes include 71 studies covering 6 domains. These domains are information and eHealth use (eg, patients’ use of the internet for pregnancy information [22]), lifestyle factors such as gestational weight gain, exercise, and smoking cessation (eg, technology-supported diet and lifestyle interventions [23]), gestational diabetes (eg, telemedicine for diabetes in pregnancy [24]), mental health (eg, therapist-supported internet-based cognitive behavior therapy among postnatal women [25]), low- and middle-income countries (eg, mobile health [mHealth] interventions for prenatal, birth, and postnatal period in low- and middle-income countries [26]), and telemonitoring and teleconsulting (eg, wireless antepartum maternal-fetal monitoring [27]). There were 16 studies related to screening and treatment for PND (van den Heuvel et al [21], 2018).

Hussain-Shamsy et al [28], 2020, aimed to understand the extent, range, and nature of mHealth tools for prevention, screening, and treatment of perinatal depression and anxiety in order to identify gaps and inform opportunities for future work. Compared to the van den Heuvel et al [21] review, it targeted interventions delivered through mobile phones, including apps and text message–based interventions with prevention, screening, and treatment purposes. Tools were for prevention (10/22, 45%), screening (6/22, 27%), and treatment (6/22, 27%). Interventions delivered included psycho-education (16/22, 73%), peer support (4/22, 18%), and psychological therapy (4/22, 18%); however, interventions that started in pregnancy and continued into the postnatal period were rare (2/22, 9%).

In the same year, Dosani et al [29] investigated existing uses of mobile phone technologies for perinatal depression in low- and middle-income countries, finding improved depressive symptoms after the interventions. Similarly, Wan Mohd Yunus et al [30], 2022, found positive outcomes for digitalized cognitive behavioral therapy (CBT) interventions for depression symptoms during pregnancy. However, it should be noted that the studies within this systematic review demonstrated relatively high risks of bias and some missing outcome data. Lau et al [31] (2022) also conducted a review synthesizing 18 randomized controlled trials and demonstrating support for the effectiveness of digital CBT for perinatal psychology symptoms (ie, depression, anxiety, and stress symptoms) in high-income countries; however, again, the included studies were found to be of limited quality. It is argued that the literature is promising in its indicative efficacy for digital and mobile interventions for perinatal mental health; however, further robust, high-quality, larger sample size, and interprofessionally co-designed randomized controlled trial research between health care professionals and application developers is required in this field.

Beyond the more common digital and mobile interventions, VR has been explored for its utility in this field. In 2024, Fallon et al [32] conducted a scoping review on the use of VR to support parents during birth and in the first year postbirth in different settings, finding that across these studies, VR was found to be effective in improving both physiological and psychological outcomes. Furthermore, mothers reported positive experiences of using VR, which could indicate VR’s acceptability in this population.

Literature has looked not only at interventions targeting symptoms but also at prevention. Some have assessed web- and mobile-based psychological interventions’ role in preventing depression during the perinatal period [33], as well as reviews on the current state of diagnostic and screening apps for perinatal mental health [34]. These review studies highlight important considerations for future research, including the need for comprehensive digital assessment tools, ensuring data protection and safety of the intended app use, and improving data sharing features between users and health care professionals for timely support.

Other prevention-focused research has aimed at increasing partner support (Pilkington et al [35], 2015). Several prevention studies that include a partner component have demonstrated some benefits [35]. However, not all of these interventions were delivered to both mothers and fathers, and research evaluating their effects on paternal mental health is lacking. Thus, it is argued that future research needs to focus more on developing active interventions for both partners.

In the past decade, numerous reviews have focused on digital interventions for perinatal mental health, particularly PND. Each of these reviews had distinct aims, ranging from examining eHealth developments during pregnancy [21] to evaluating the effectiveness of mHealth tools [28] and digital CBT interventions [30]. Others, such as Fallon et al [32], explored emerging technologies such as VR for supporting parents during the postnatal period. These reviews covered a variety of tools and populations, including both high- and low-income countries, and targeted different stages of perinatal care. Efficacious tools are important. However, those with perinatal mental health symptoms have demonstrated delays in seeking those interventions [36]. To the best of the authors’ knowledge, there are limited review papers focused on how digital interventions can improve help-seeking behaviors in perinatal depression, highlighting a significant literature gap with important clinical and economic implications for undertreatment.

There is a paucity of research that explores how the games are designed for PND. However, there is a game-specific exploration of depression symptoms more generally, which is of relevance given that PND and major depression share identical symptoms except for onset [37]. In 2014, there were 2 relevant review papers. One is the systematic review by Fleming et al [38] of the evidence for serious games in the treatment or prevention of depression. The current data suggest that it is possible to develop serious games for depression, that young people are willing to try them, and that available adherence and impact data, whilst limited, are promising. The other systematic review is Li et al [39] systematically examining the effectiveness of game-based digital interventions for depression, investigating psycho-education and training, VR Exposure Therapy, exercise, and entertainment. Psycho-education and training and VR Exposure Therapy were identified as the most popular types of game applications for depression. Given the demonstrated potential gains for global access, it becomes even more crucial to meet the needs of those reluctant to seek help through current methods.

To the best knowledge of the authors, there is only one relevant review from 2018 (Dias et al [40]). Dias et al [40] aimed to identify through a systematic review how gamification and serious games support depression treatment and identified two significant literature gaps, including the limited study of (1) the effectiveness of gamification and serious games for depression and (2) hazard assessment of the side effects of using gamification and serious games in depression treatment. The technologies identified in this review included mobile, computer, wearable, and web applications that were applied in gamification, serious games, VR, and speech analysis. This area has not been revisited since this 2018 review, as far as the authors are aware, yet in recent years, there has been an increasing interest in serious games for depression and health problems more broadly [41].

A systematic review of casual video games (CVGs) by Pine et al [42] provided a systematic review of the effects of CVGs on treating anxiety, depression, stress, and low mood. This work includes 9 CVGs, of which 6 studies aimed at reducing anxiety, 2 studies examined effects for depression, and 4 studies investigated the impact of CVGs on treating stress or low mood. CVGs are becoming increasingly popular, and people report playing them for various reasons, such as relieving stress and relaxing, which may provide some initial benefit for those who need additional support or are waiting to access more comprehensive treatment.

In 2021, a total of 4 relevant works on games for depression and border mental health concerns were published in Australia, Ireland, Austria, and Taiwan. King et al [43] narratively reviewed indie—or independent—games that address mental health, trauma, and grief, highlighting developers’ difficulties due to insufficient game design and research studies when creating, developing, and evaluating serious games. Indie games were initially defined as games made on low budgets by small teams that were published outside more mainstream channels and used by larger companies. There are many challenges when developing serious games, including limited funding, small teams, a need for broad expertise, and limited examples for developing both games and studies. Serious mental health games that target those with mental illness directly face additional challenges, such as mental health disorder symptoms that can affect the present ability to engage with content. This challenge means developing mental health serious games presents unique challenges to developers, who must decide how to best address their intended audience.

Differing from previous work that targeted independent games, Kowal et al [41] focused on the mental health benefits associated with playing commercial video games to address symptoms of depression and anxiety. In light of the current research, we conclude that commercial video games show great promise as inexpensive, readily accessible, internationally available, practical, and stigma-free resources for mitigating some mental health issues in the absence of, or in addition to, traditional therapeutic treatments. Aside from the game target general public, Martinez et al [44] investigated serious games published between 2015 and 2020 for depression and anxiety in children and adolescents, with a new approach focusing on their applications: awareness, prevention, detection, and therapy. The results of this systematic review show that more awareness and detection games and games with awareness, prevention, detection, and therapy applications are needed. In addition, games for depression and anxiety should equally target all age ranges. For future research, developing and evaluating serious games should be standardized, and the games should always offer support while playing. Meanwhile, Yen and Chiu [45] conducted a review to explore the effectiveness of VR exergames in improving older adults’ cognition and ameliorating depressive outcomes. This study suggests that VR exergames can potentially positively influence cognition, memory, and depression in older adults. VR exergames could be an interesting strategy for active aging and a good mental health status.

In 2022, there were 5 key sources. First, Abd-Alrazaq et al [46] aimed to assess the effectiveness of serious games in alleviating depression by summarizing and pooling the results of previous studies. Their findings indicate that exergames are as effective as active interventions, which are usually delivered and supervised by health care providers. Meanwhile, Ruiz et al [18] systematically reviewed the evidence and found video game-based interventions were valuable and effective in reducing symptoms of depressive disorders. Additionally, Kim et al [47] conducted a systematic review, finding that serious games were beneficial in reducing depression in older adults. Regardless of the study setting, serious games appeared to reduce depression. Particularly, serious games, including physical activities, had a significant impact on reducing depression.

Concerning combined depression and anxiety-related symptoms, Townsend et al [19] conducted a review into the effectiveness of gaming interventions for treating either depression or anxiety in individuals aged 12‐25 years. Preliminary evidence suggests that gaming interventions may be an effective treatment for youth depression but not anxiety. Further research is warranted to establish the utility, acceptability, and effectiveness of gaming interventions in treating mental health problems in young people.

The fifth relevant work of this year was published by Chitale et al [48], reviewing the viability of games and VR for the assessment of anxiety and depression, finding that possible digital correlates or biomarkers of depression and anxiety could help researchers with their design. It is important to emphasize that to ensure safety, efficacy, and privacy at a health care standard. It is argued that game developers and researchers must collaborate interprofessionally with qualified mental health specialists. More clinical data is necessary to further evidence the effective use of video games or VR in assessment methods for anxiety and depression.

In 2023, Gliosci et al [49] systematically reviewed the current array of scientific research on video games used as a therapeutic intervention tool for depression. The latest research on the use of video games as depression treatment methods shows that there are significant gaps in the sector, particularly across generations. The age group that is most affected by the condition and now consumes the most video games—adults aged 18‐40 years—has had limited focus in the game and mental health literature. Given that depressed people tend to be more isolated, there may be untapped possibilities, such as the unique way that games can bring people together, that could be used in different types of interventions.

Most recently, in 2025, Gómez-León [50] systematically reviewed the current serious games designed to train emotional regulation skills in children and adolescents, finding that serious games can be effective, acceptable, and feasible for learning emotional regulation strategies and reducing symptoms related to depression, anxiety, and lack of impulse control.

As highlighted, the current reviews have several limitations and clinically significant research gaps that need to be addressed. Existing reviews rarely address digital interventions specifically targeting parents in late pregnancy or postnatal stages. Most studies focus on adolescents, older people, or the general population with depression. Given the unique physiological and psychological needs of a perinatal mental health group and the common reluctance to seek help, it is crucial to develop targeted digital games and help-seeking interventions for new parents, especially during late pregnancy and the postnatal period.

Current reviews focus primarily on symptom relief rather than fostering help-seeking behaviors. For new parents, it is essential to explore how digital games can educate and provide psychological support to help them recognize symptoms of depression and empower them to seek professional help. Crucially, the physiological and psychological changes that occur during pregnancy and the postnatal period differ from those experienced by the general population with depression, often influenced by hormonal shifts and changes in life roles [1]. Existing reviews lack a deep exploration of how these specific mechanisms impact the effectiveness of gamified interventions, and therefore, this will be a particular aim of this review. Game design must consider these unique psychological and physiological changes to support the mental health of new parents effectively.

Although serious games have shown promise in supporting mental health, few have been tailored to the unique needs of new parents facing PND. Existing interventions often overlook practical constraints such as time limitations and emotional vulnerability. Given this need, the current review aims to explore how game-based tools can be thoughtfully designed to provide emotional support, foster help-seeking, and offer accessible education during this critical period.

There is growing recognition that serious games could offer an accessible, flexible, and emotionally supportive solution for broad mental health problems. This narrative review provides a comprehensive and interpretative narrative synthesis of the existing literature on serious games and digital interventions for PND and, where data is limited, extends its review to related literature on depression, given its almost identical diagnostic profile with PND. The aim is to critically assess prevailing trends and gaps and to inform the need for targeted research in key, often overlooked, areas. By addressing these aspects, we aim to make future direction recommendations for research and game developments that could cater to the unique needs of parents in late pregnancy and postnatal stages, particularly in raising awareness of help-seeking behaviors toward PND. This review aims to highlight how digital games can be more effective in supporting this specific underserved population.

## Methods

### Search Strategy and Study Selection

The literature search was conducted across 7 research databases and publisher repositories: PubMed Central, the ACM Digital Library, Google Scholar, the IEEE Xplore Digital Library, Science Direct, the Springer Library, and Wiley. Keywords included combinations of major terms such as “serious games,” “digital games,” “postnatal depression,” and “help-seeking.”

The resulting search string is as follows and shown in Table 1: ((serious game OR serious games OR gamification OR gamified OR game design OR game based OR game-based OR gaming) AND (postnatal depression OR postnatal depression OR perinatal depression OR maternal depression OR post-birth depression)). For the detailed search strategy, please refer to Multimedia Appendix 1.

**Table 1.** Search string.

Major terms	Synonyms
Postnatal depression	postnatal depression OR perinatal depression OR maternal depression OR post-birth depression
Serious game	serious games OR gamification OR gamified OR game design OR game based OR game-based OR gaming

The search covered literature published between 2015 and 2024 and was limited to studies published in English. We chose to only include articles published after 2015, considering relevance and time efficiency.

Given the only 2 game research specific to PND, we also chose to include depression as an analogous disorder for inclusion because, according to the *DSM-5* (*Diagnostic and Statistical Manual of Mental Disorders*, Fifth Edition) [51], major depressive disorder and PND share core diagnostic symptoms. Thus, the broadened search string is as follows: ((serious game OR serious games OR gamification OR gamified OR game design OR game based OR game-based OR gaming) AND (depression OR sadness OR depressive))

Initial search results were screened based on titles and abstracts by author WW. Then, full-text reviews were conducted by author WW and decided across the team, EP and JG, to determine their relevance to the research objectives. Reference lists of key articles were also reviewed to identify additional sources. The screening process is shown in the Results section.

Studies were included if they discussed the design, application, or evaluation of serious games related to promoting help-seeking behaviors toward depression or perinatal mental health. Articles focusing solely on maternal health without mention of game-based interventions were excluded.

### Sufficiency Statement

The authors acknowledge that their disciplinary backgrounds and research interests in digital mental health and game design may have influenced the interpretation and prioritization of specific themes during the analysis. Author WW is a PhD candidate who has experience in interaction design, data analysis, and software development. Author EP is a senior lecturer in the faculty of health. She has published reviews and empirical research in the field of mental health. She is also a clinical psychologist practitioner working with vulnerable and mentally unwell clients and families, including clients with PND. Author JG is a senior lecturer in games development, an experienced software engineer, and a serious game designer working with diverse clients. A particular emphasis was placed on design elements that support emotional engagement and help-seeking behaviors, which aligns with the authors’ focus on developing interventions for new parents experiencing PND. Additionally, the narrative nature of the review allows for interpretive flexibility, which, while beneficial for exploring emerging ideas, may introduce subjective bias in theme identification and synthesis. The selection of literature in English only and a focus on peer-reviewed sources may have also limited the inclusion of culturally diverse perspectives and nontraditional forms of evidence.

### Analysis Approach

To guide the synthesis and interpretation of findings across the selected literature, this review adopted a thematic analysis approach. Commonly used in qualitative research, thematic analysis is a systematic method for identifying, analyzing, and interpreting patterns of meaning—referred to as themes—within textual data [52]. It is particularly appropriate for narrative reviews, as it allows for the integration of diverse sources and offers a deeper understanding of complex issues across varied contexts. The analysis followed the 6-step process [53]. Initially, all included texts were read thoroughly by author WW to ensure familiarity with the content. From there, initial codes were generated inductively by author WW to capture recurring ideas, concepts, and relevant patterns. These codes were then reviewed by the team (EP and JG) and grouped into broader themes that reflected meaningful trends across the dataset. Each theme was refined and decided across the team (WW, EP, and JG) to ensure clarity, distinctiveness, and relevance to the review’s aims. The final step was author WW weaving the themes into a cohesive narrative that highlighted their significance in relation to the central research questions.

By using thematic analysis, this review was able to go beyond a simple summary, offering a structured yet flexible framework to interpret findings. A thematic narrative approach was considered appropriate due to the heterogeneity of the sources, which varied in methodology, population focus, and design strategy. This flexibility allowed for a deeper exploration of how serious games function as educational and emotional tools, especially in the context of PND.

## Results

### Overview

**Figure 1.**
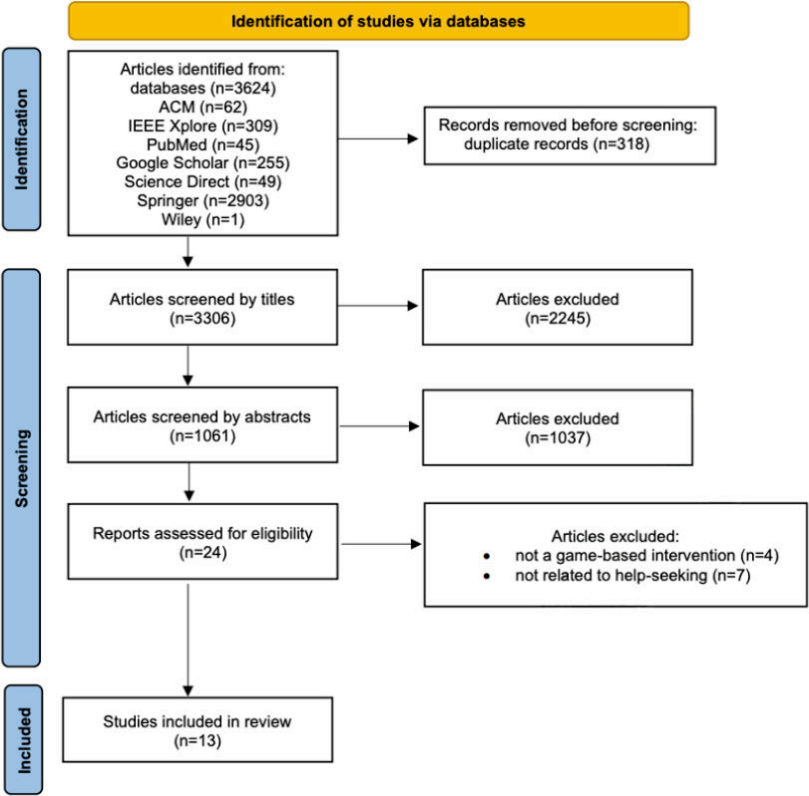
PRISMA diagram. PRISMA: Preferred Reporting Items for Systematic Reviews and Meta-Analyses.

There are 2 studies identified specifically from the search string PND, and 11 studies included from the search with depression. In total, 13 eligible studies were included in this review. There were 9 games published in academic articles, and the other 4 are from market research. In the past 5 years, there were 5/9 (55.6%) studies published. The screening process is shown in Figure 1. An overview of their characters is shown in Table 2.

**Table 2.** Summary of included studies.

Name	Year	Country	Study design	Sample size	Intervention length
Above Water	2016	Canada	N/A^a^	N/A	N/A
Stigma-Stop	2017	Spain	Comparison study	552	N/A
Leadership Training in Mental Health Promotion (LMHP)	2017	Germany	Quasi-experimental study	48	3 months
Moving Stories	2019	Netherlands	Cluster randomized controlled trial	185	6 months
MindMax	2020	Australia	Participatory design	40	N/A
MANTRA	2020	United Kingdom	Cocreation process	60	N/A
Hellblade: Senua’s Sacrifice	2020	United States	Two-condition, randomized design	198	45 minutes
COEX-IST	2022	Brazil	N/A	N/A	N/A
PPDHero	2024	Bangladesh	Requirement elicitation study	108	N/A

a N/A: the data were not clear or not mentioned in the original paper.

This section divides the 13 studied games included into 3 categories based on their perspectives on improving help-seeking behaviors: promoting knowledge, reducing stigma, and raising awareness.

### Promoting Knowledge

#### Overview

An overview of the games that focus on promoting knowledge to help improve depression can be seen in Table 3.

**Table 3.** Games focused on promoting knowledge.

Name	Year	Category	Aim	Audience	Game engine	Player mode
Above Water [54]	2016	Strategy games	This game informs people about the available strategies to cope with 2 types of anxiety disorders—generalized anxiety disorder and panic disorder. The game teaches players about existing treatments.	Game players	Physical cards and a mobile application	Multiple players
Moving Stories [55]	2019	RPGs^a^	This game aims to make adolescents have better mental health literacy and endorse fewer stigmatizing attitudes regarding depression.	Adolescents	Not specified	Single player
MindMax [56]	2018	Casual games	This game is to deliver psycho-educational modules and create a web-based community centering on well-being, AFL^b^.	Men aged 16 to 35 years who are interested in AFL or video games	Mobile phone	Single player
MANTRA [57]	2020	Casual games	This game is to increase maternal and child health resilience before, during, and after disasters.	Vulnerable low-literacy female audiences in rural Nepal	Mobile phone	Single player
PPDHero [58]	2023	Serious games	This game aims to screen PND^c^ and provide education and support to new mothers to identify and manage symptoms and connect with health professionals.	New mothers	Mobile gamification app	Single player

a RPG: role playing game.

b AFL: Australian Football League.

c PND: postnatal depression.

#### Above Water

In 2016, Wehbe et al [54] released the educational game *Above Water* for anxiety. It is a digital or physical hybrid game to inform people about the available strategies to cope with 2 types of anxiety disorders, which are Generalized Anxiety Disorder and Panic Disorder. It is designed to inspire players to share their experiences and develop their narratives by teaching them existing treatments. *Above Water* is a game that players play with physical cards and digital devices, such as tablets and smartphones. The game is designed to be played with 4 players. They are required to achieve all 6 categories of life goals, including career, education, health, self-improvement, financial stability, and relationships, while managing anxiety. They need to select 4 life goals on their smartphone, which cannot be changed during the play session. On each player’s turn, they will draw 1 card to face up, play, or discard. Anxiety cards can be discarded by using treatment cards. They can be taught deep breathing skills, yoga, and stretching poses using mobile applications. The digital implementation does not require downloading an app. It uses a simple HTML5-enabled website. This game has several strengths, particularly in raising awareness and encouraging social support through collaborative gameplay. Its focus on educating players about anxiety and introducing treatment options makes it helpful in fostering an open dialogue about mental health. However, the game falls short in targeting specific populations and providing tailored interventions. Additionally, it lacks mechanisms for improving long-term behavior change and cultural adaptation, which are critical elements for effectively addressing the unique challenges faced by parents dealing with PND. It could be improved by incorporating specific content for new parents and addressing unique social and cultural barriers.

#### Moving Stories

*Moving Stories* is a game-based school program for mental health literacy and stigma regarding depression published by Tuijnman et al [54] in 2019. It is developed by a professional game design company in collaboration with researchers and relevant stakeholders. Moving Stories includes three parts: (1) an introduction lesson, (2) a single-player, mobile, 3D video game, and (3) a contact session with someone with lived experience with a depressive disorder. The program focuses on three perspectives of mental health literacy: (1) recognizing the disorder, (2) having knowledge of help-seeking options and treatment available, and (3) first aid skills to support others who are developing or in a mental health disorder. The video game asks players to help a girl named Lisa, who has almost lost interest in everything. The players can do multiple things for Lisa to accumulate or decrease their relationship scores. As a bridge, the players are guided to share messages and discuss the reflection in a contact session with the school welfare coordinator or school counselor. This game demonstrates several strengths as a mental health intervention tool, particularly in improving mental health literacy, reducing stigma, and promoting help-seeking behaviors. By empowering players with knowledge, its approach to directly confronting myths through gameplay helps break down stigma. It encourages a more open conversation about mental health issues, which sets the foundation for increased understanding and empathy. It is crucial to tackle the stigma that new parents might face regarding PND. A key strength of the game lies in its focus on promoting help-seeking behavior. Through interactive scenarios that involve assisting the character Lisa and guiding her toward appropriate support, *Moving Stories* emphasizes the importance of recognizing mental health issues and seeking help. This game aligns well with the need to address the avoidance of help-seeking behaviors seen in PND, where new parents often struggle to ask for support due to stigma or lack of awareness. Incorporating role-playing elements demonstrates how and when to seek help, which is critical for empowering individuals to take action in real-life situations.

#### MindMax

*MindMax* is an app that incorporates gamification, mini-games, and social connection to improve men’s mental health and well-being, published by Cheng et al [56] in 2018. This game is based on the original well-being training program that the Australian Football League Players’ Association offered players. Thanks to the collaboration with the University of Sydney and the University of Technology Queensland, it aims to deliver a portable, digital version of Australian Football League Players’ Association’s existing program. The design process includes 3 phases. The first phase is 6 participatory design workshops consisting of 3 stages: discovery, evaluation, and prototype to identify how best to frame the well-being concept discussed in *MindMax* and, more broadly, how to structure a mental health and well-being app for the intended audience. The second phase is knowledge translation. The third phase is user experience testing interviews. This work explained well why and how they implement the participatory design process. The core components of *MindMax* include two parts: (1) multiple educational modules, each around 10 minutes, and (2) a web-based community centering on well-being, sports, and video games. The good point in an educational context is that the audience does not care about what context is presented, but how it was presented. The tone of expression is very important. Regarding the social element, it suggested the app should enable rather than emulate, and sharing personal experiences with potential groups they are conscious of may need careful consideration.

One of the key strengths of *MindMax* is its incorporation of psycho-educational modules that provide users with mental health information in a clear and accessible manner. Each module is designed to be short (approximately 10 min), making it feasible for users with limited time, such as new parents with demanding schedules. These modules cover foundational topics such as mindfulness, emotional well-being, and basic coping skills. For new parents experiencing PND, such content can be particularly beneficial as it provides them with the tools they need to understand their emotional state and learn practical strategies for managing stress. Making these modules easily digestible helps ensure that even those unfamiliar with mental health concepts can engage with the material effectively. Besides, the mini-games and reward-based mechanics used in *MindMax* are particularly useful in maintaining user engagement, which might be a crucial factor for individuals who may be overwhelmed with responsibilities or have limited energy to devote to self-care. Using games as rewards helps create positive reinforcement for completing educational tasks, encouraging continued learning and interaction. For new parents, especially those struggling to make time for themselves, gamification can transform mental health support into something enjoyable and less intimidating, thus promoting consistent use of the intervention. The light-hearted games and achievement-based systems can provide much-needed moments of relaxation and enjoyment, breaking up the often stressful and exhausting routines of caring for a newborn.

#### MANTRA

*MANTRA* is a serious mobile game that aims to reach a low-literacy audience in a low-resource setting with knowledge of maternal health, neonatal health, and geohazards. This work was published by Mueller et al [57] in 2020. The game mechanic is relatively simple in this game: picture matching with audio and visual feedback. The project’s core goal is to tailor the knowledge to a local cultural context to fit specific settings with low literacy and resources. Throughout the design process, co-design and cocreation were guiding principles. The game design methodology builds on these principles and typical software development processes. The “drag-and-drop” is the only mechanism needed in the game. There is a touchscreen tutorial at first to help users who are not familiar with the screen interface. There are 3 modules in total, each containing 3 levels of complexity. The unsuccessfully answered question will be pulled into an error pool and repeated up to 3 times throughout the level. The player will fail this level if all 3 times are answered incorrectly. *MANTRA* demonstrates several notable strengths in health education, particularly its accessibility for low-literacy users, cultural localization, and reinforcement of knowledge through repetition. It is designed for low-literacy users. It uses pictograms, audio prompts, and simple, intuitive interactions to ensure that even those with limited literacy skills can engage with and benefit from the game. This setting is particularly valuable for new parents from underserved communities who may lack access to formal health education, effectively addressing knowledge gaps. Second, the game’s cocreation and cultural localization enhance its relevance and acceptance. Given that cultural context plays a significant role in the understanding and treatment of PND, incorporating cultural elements into the game helps to establish a deeper connection with users, reduce stigma, and increase engagement. Lastly, *MANTRA* uses repeated learning modules and immediate feedback, which helps reinforce knowledge retention. For new parents needing to master coping strategies and understand when to seek help, this method of repetition is crucial for effectively supporting their mental health outcomes. However, *MANTRA* also has some limitations. First, while using simple mechanics (such as drag-and-drop) makes the game accessible, it may not provide the depth of engagement needed for users facing significant mental health challenges. Second, the game lacks features that actively promote help-seeking behavior. For a digital game aimed at addressing PND, it is essential to include explicit pathways to professional support, such as information on local health care services or in-game guidance on seeking help, to bridge the gap between awareness and action. Lastly, the content of *MANTRA* primarily focuses on mothers without sufficient consideration of the role of fathers or nonbirthing parents in supporting maternal mental health.

#### PPDHero

*PPDHero* is a novel web-based system published by Sara et al [58] in 2024 for new mothers to screen PND and provide them education and support to identify and manage symptoms and connect with health professionals if needed. The design process starts with an elicitation study, which involved 6 senior doctors, who were incorporated with surveys from the broader public. The system was developed based on interview and survey data analysis. It includes three modules: (1) relevant content management; (2) screening, assessment, and gamification; and (3) a treatment and support module to enable users to share resources, enroll in screening procedures and treatment plans, register for gamified activities, and involve trusted professionals in their postnatal journey. The gamification tasks, such as quizzes and exercises, are implemented in the screening and assessment session to enhance the assessment process. Its strengths lie in its comprehensive approach, integrating education, screening, and treatment in a single platform. Using gamification elements, such as quizzes and interactive activities, enhances user engagement and makes learning about PND more attractive. Additionally, the platform provides a personalized experience through tailored treatment plans based on screening results, ensuring that users receive targeted support to manage their mental health. However, while the platform provides education and support, it lacks features specifically aimed at reducing stigma, which is an important factor in encouraging new mothers to seek help.

### Reducing Stigma

#### Overview

In Table 4, there is an overview of game-related components that contribute to depression and broad mental health illness by reducing stigma to the game players.

**Table 4.** Games focusing on reducing stigma.

Name	Year	Category	Aim	Audience	Game engine	Player mode
Stigma-Stop [59]	2017	Simulation games	This game aims to reduce the stigma toward mental illnesses.	High school students	Unity3D	Single player
Leadership Training in Mental Health Promotion (LMHP) [60]	2017	Serious games	This game is to promote employee mental health and reduce mental illness stigma at work.	Managers	Digital game-based training program	Single player
Hellblade: Senua’s Sacrifice [61]	2020	RPGs^a^	This game aiming to let players play video games featuring characters enduring mental illness may ultimately reduce stigma through transportation and identification.	Game players	Unreal Engine 4	Single player

a RPG: role playing game.

#### Stigma-Stop

*Stigma-Stop* is a 3D serious game to reduce the stigma toward mental illness among high school students, published by Cangas et al [59] in 2017. It is specifically designed to reduce the stigma associated with mental health disorders by educating players about different mental health conditions, such as depression, schizophrenia, bipolar disorder, and panic disorder with agoraphobia. The game provides information about these disorders through interactive dialogues and scenarios where players must choose how to react to individuals with mental health issues. This kind of approach is effective in challenging and correcting misconceptions about mental illness, particularly among young people. The game presents players with scenarios of interacting with characters representing different mental health conditions. Players are asked how they perceive the characters’ psychological state, whether they have experienced similar emotions, and how they can help the character. These choices, along with the feedback provided for correct and incorrect responses, allow players to learn more about mental health hands-on, engagingly. However, the game also has notable limitations. Its primary focus on adolescents and broader mental health disorders may make it less directly relevant for new parents dealing specifically with PND. Additionally, the game lacks practical coping strategies or therapeutic guidance, which limits its effectiveness as a support tool aimed at improving the help-seeking behaviors of PND.

#### Leadership Training in Mental Health Promotion (LMHP)

*Leadership Training in Mental Health Promotion (LMHP)* is a digital game-based training program for managers to promote employee mental health and reduce mental illness stigma at work, published by Hanisch et al [60] in 2017. It applied various gamification components, such as providing a storyline and clear goals, including the capacity to overcome challenges by learning, giving feedback on performance, showing progress, and reinforcing learning by allocating points rather than simply providing badges for achievements and enabling competition between players, to facilitate an innovative and engaging learning experience. *LMHP* demonstrates several strengths that make it an effective tool for supporting mental health in the workplace and reducing stigma. The program’s focus on mental health literacy, reducing stigma, and improving self-efficacy among managers makes it well-suited to foster a supportive work environment. The interactive simulation-based approach helps participants practice these skills in a safe environment, leading to higher engagement and better skill retention. While *LMHP* has strengths in terms of mental health literacy and stigma reduction, it focuses on the workplace environment and specifically targets managers. The scenarios and skills taught are tailored to workplace situations, which may not fully address new parents’ specific emotional and psychological challenges in their home environment.

#### Hellblade: Senua’s Sacrifice

*Hellblade: Senua’s Sacrifice* is an action-adventure game accurately portraying psychosis to reduce public mental health stigma, developed and published by Ninja Theory [61] in 2017. It aims to reduce the mental health stigma in 2 ways: by lowering stereotyping and limiting participants’ desire for social distance. *Hellblade: Senua’s Sacrifice* is set in an age of Vikings. Its story is about Senua’s battles for the soul of her departed lover, Hela. Senua endures psychosis and must contend with her mental illness along with the challenges presented by her quest. The game blends gameplay mechanics and concepts such as puzzle solving, psychological horror, and melee combat. Voice acting is an integral part of the game, while its cut scenes combine motion capture by Melina Juergens and live-action performances by other actors.

The game’s powerful and immersive portrayal of mental health struggles provides players with a unique opportunity to experience the emotional complexities associated with conditions such as psychosis. Through this kind of realistic depiction, new parents experiencing PND can see their struggles mirrored in the protagonist’s experiences, helping them feel validated and understood. Second, the game’s high-quality audio design plays a crucial role in enhancing the emotional depth of the experience. Using binaural audio to simulate auditory hallucinations, *Hellblade: Senua’s Sacrifice* creates a profoundly immersive atmosphere that draws players into Senua’s inner world. This kind of powerful audio experience can help new parents connect more profoundly with the emotional struggles portrayed, fostering empathy and offering a unique perspective on mental health challenges. Lastly, the game’s development process involved collaboration with mental health experts and individuals with lived experience of psychosis. This process makes the portrayal of Senua’s mental health struggles both vivid and respectful, adding credibility to the game’s narrative.

### Raising Awareness

#### Overview

In Table 5, there is an overview of how game-related components contribute to depression and broad mental health illnesses by raising awareness among game players.

**Table 5.** Games focused on raising awareness.

Name	Year	Category	Aim	Audience	Game engine	Player mode
Depression Quest [62]	2013	RPGs^a^	This game aims to show other people with depression that they are not alone in their feelings and to illustrate to people who may not understand the illness the depths of what it can do to people.	Game players	Twine engine	Single player
Keep in Mind: Remastered [63]	2018	Simulation games	This game is meant to be a therapeutic experience for those who struggle with mental illness on a journey of reflection and emotional healing.	Those who struggle with mental illness or those who find themselves lost in the dark	Unity	Single player
Before I Forget [64]	2020	Simulation games	This game aims to raise awareness about mental health and communicate to the audience the struggles of people experiencing dementia.	Game players	Unity	Single player
Sea of Solitude [65]	2021	Simulation games	This game aims to reveal the themes of depression, loneliness, and hopelessness, how we unwittingly push people away, and how our actions can negatively affect others.	Game players	Unity	Single player
COEX-IST [66]	2022	Strategy games	This game is meant to increase mental health awareness and focus on self-care as a form of prevention.	People with a wide age range, from teenagers to adults	Not specified	Single player

a RPG: role playing game.

#### Depression Quest

*Depression Quest* is an interactive fiction game dealing with depression. It was developed by Zoe Quinn [62] using Twine (Interactive Fiction Technology Foundation) and published in 2013. This game aims to spread awareness by showing other people with depression that they are not alone in their feelings. *Depression Quest* is not an easy gaming experience because its goal is precisely to let you experience the difficulties of depressed people. It is a text-based game, and your choice will impact your ending. *Depression Quest* has a straightforward game structure. There are no animations or complex controls. As the story progresses, the player chooses different options to move the story in different directions. The part that *Depression Quest* comes closest to reality is that it tries to make people who do not have experience with depression understand that there are choices that do not exist for people who have depression. In the game, as the depressive condition deepens, the optimistic options are crossed out by the system with a red line—the player can only choose the closed, pessimistic options, and the depressive condition further deepens. There are fewer and fewer options to choose from in the game. It is a vicious circle and one that many people with depressive tendencies face.

This game has several strengths in its design elements. First, the game’s narrative-driven depiction of depression provides a deeply empathetic look into the daily struggles of a person experiencing mental health challenges. Second, the game uses a unique mechanic where certain positive choices become restricted based on the protagonist’s mental health status. This mechanic reflects the real-life constraints imposed by depression, such as the difficulty in making proactive decisions when feeling overwhelmed. For new parents experiencing PND, this feature can help explain why even seemingly simple actions might feel impossible. It encourages self-compassion by showing that these limitations are part of the condition, not personal failings. Furthermore, as players engage with the narrative and take actions such as seeking help, they unlock new options, which effectively illustrate the empowerment that can come from actively addressing mental health challenges. Third, *Depression Quest* is also effective in raising awareness and fostering empathy among those who may not have firsthand experience with depression. By taking players through scenarios involving hopelessness, lack of motivation, and interpersonal struggles, this game mechanic could help partners and family members of new parents better understand the challenges their loved ones face. This improved understanding can lead to more supportive relationships and foster a compassionate environment. Finally, the game places a strong emphasis on encouraging help-seeking behavior. Players can pursue therapy or medication throughout the match, impacting the protagonist’s situation and improving the choices. By showcasing how seeking help can positively affect mental health, *Depression Quest* actively promotes the idea that professional support is valuable and effective. New parents, who may feel hesitant to seek help due to stigma or uncertainty, could see the benefits of reaching out in this kind of game, which can help motivate them to pursue the support they need.

However, despite these significant strengths, the game has a key limitation. The intense focus on the struggles of depression, combined with the dark and sometimes overwhelming themes, may risk leaving players feeling disheartened without enough positive reinforcement or hope to balance the experience.

#### Keep in Mind: Remastered

*Keep in Mind: Remastered* [63] is a heavy story-driven psychological indie game that follows Jonas, a man who struggles with grief, depression, and alcoholism, on a journey of reflection and emotional healing. One night, he awakens to a shadowy mirror world where beasts lurk, and stars do not shine. Lost and scared, Jonas must face the twisted beasts if he ever wishes to return home and learn the truth about his darkness. It was released by Little Moth Games and Akupara Games in March 2018. This game was created for those who struggle with mental illness or those who find themselves lost in the dark. This game is meant to be a therapeutic experience. *Keep in Mind: Remastered* has notable strengths that make it a compelling game for raising awareness about mental health challenges. For new parents, especially those experiencing PND, the portrayal of such internal struggles can help them feel seen and understood. This level of emotional storytelling can help reduce the stigma around PND by showing that struggling with mental health issues is not uncommon and that others share the negative thoughts and emotions they may be experiencing. Besides, the game uses symbolism effectively to represent the different facets of the protagonist’s mental health challenges, such as dark creatures and eerie settings. This symbolism allows players to externalize their internal emotional struggles, making them more tangible and easier to understand. However, the game’s focus on heavy and potentially triggering themes, lack of practical coping strategies, and absence of integrated help-seeking features might limit its effectiveness as a supportive intervention.

#### Before I Forget

*Before I Forget* is a single-player exploration game developed and published by 3-Fold Games [64] in 2020. It is a short game that draws the player to a character experiencing dementia. She is housebound and unable to leave the flat. Only the windows could offer a view of the outside world, with its letterboxes and birdbaths. Otherwise, the exploration all happened in the mind as the character scrabbles to dredge artifacts from her past. In this game, players guide Sunita Appleby, who is a scientist with early-onset Alzheimer disease. As she interacts with now-unfamiliar objects in her house, some of her memories return. The game features puzzle-like elements, such as recalling where mementos have been left and asking players to guide Sunita to the bathroom when she perceives her house as a shifting maze. The game’s use of empathetic storytelling and emotional depth provides a powerful connection to the struggles faced by those with mental health challenges. The game fosters understanding and reduces stigma around vulnerability and emotional struggle by allowing players to see the world through the protagonist’s perspective. Additionally, the emphasis on relationships and emotional connections highlights the importance of support networks, encouraging players to lean on their loved ones during difficult times. However, the game also has notable limitations; the lack of practical coping strategies limits its effectiveness as a tool for managing day-to-day mental health issues, such as PND.

#### Sea of Solitude

*Sea of Solitude* is an adventure video game developed by Jo-Mei Games and published by Electronic Arts [65]. The player controls a young woman named Kay, who endures such strong loneliness that her inner feelings of hopelessness, anger, and worthlessness turn to the outside, and she becomes a monster. As Kay, the player explores a seemingly empty flooded city and interacts with its scaly red-eyed creatures to reveal why she turned into a monster. Her emotions manifest into giant monsters standing in her way, trying to help but also destroy her. She needs to interact with and understand their underlying intentions to overcome the negative effects of those emotions. The game is an inner dialogue of a person trying to reconcile her shortcomings. It provides an insightful look at how mental illness devastates the lives of not just those it affects but also loved ones on the outside. Kay learns a lot about herself by understanding the value of listening, coming to terms with her flaws, and not just empathizing with her family but also accepting that a simple fix is not always possible. The game’s powerful narrative effectively depicts the emotional struggles of loneliness, depression, and anxiety, allowing players to relate deeply to the protagonist’s journey. Referring to this representation can help new parents normalize their emotions and reduce the stigma around experiencing mental health challenges during the postnatal period. The exploration mechanics and emotional expression throughout the game provide a meaningful way for players to reflect on their experiences. Additionally, the game’s focus on empathy and understanding different characters’ struggles helps to foster a deeper connection between players and the themes of mental health. For new parents, this emphasis on empathy can reduce feelings of isolation and highlight the importance of reaching out for support, both for themselves and others. However, *Sea of Solitude* also has notable limitations in addressing PND effectively. The game lacks practical coping strategies that can be directly applied to real-life challenges. For new parents, having evidence-based tools and actionable advice would be instrumental in managing the day-to-day reality around PND. Without such strategies, the game may fail to deliver tangible help beyond emotional understanding. Moreover, the heavy themes of depression, anxiety, and loneliness may be overwhelming for some new parents, particularly those who are already vulnerable. Without sufficient pacing options or content warnings, the game risks triggering or intensifying negative emotions rather than alleviating them.

#### COEX-IST

*COEX-IST* is a 3D interactive story decision-making game that aims to grow awareness about depression and social isolation after a pandemic. In 2022, Rodrigues et al [66] developed and published it. The game story is based on 25 undergraduate students’ personal experiences. It is a third-person short game based on narratives and point-and-click to interact with the scene’s objects and make decisions. The end of the narrative has 2 possible actions for game over: victory over depression or victory of depression. The story’s resolution also involves tying up the loose ends of the climax and falling action. Each choice has impact consequences. Both possible ending scenes allow the player to experience and think about depression, growing awareness of this illness. The game’s interactive decision-making process, emphasis on empathy, and reflective storytelling structure offer a deep and meaningful engagement that helps players connect with the content and understand the complexities of mental health challenges. First, the interactive decision-making feature allows players to experience the consequences of their actions realistically. This level of interactivity encourages new parents to see the potential impact of small, positive actions in their own lives, thereby fostering a sense of control over their mental well-being. This sense of agency is particularly important for new parents, who may often feel overwhelmed by the responsibilities and pressures of caring for a newborn. Second, the game’s emphasis on mental health awareness and emotional empathy is effectively conveyed through storytelling. By representing depression as “Mr. Shadow,” *COEX-IST* provides a powerful visualization of the weight and burden of mental illness. This representation helps players externalize and better understand their own struggles. It also reduces the stigma associated with these emotions by demonstrating that they are a shared experience that can be overcome with the right actions. This empathetic storytelling can provide comfort and connection for those who may feel isolated in their struggles. Lastly, the structured storytelling approach gives the narrative a clear and engaging emotional experience that mirrors the journey of dealing with mental health challenges. For new parents, seeing the protagonist’s ups and downs depicted in a structured way can help normalize their own experiences. The 2 possible endings—either overcoming depression or being overtaken by it—underscore the importance of the player’s choices and illustrate the ongoing nature of mental health management. This setting highlights that while setbacks may occur, progress is possible, which can provide hope and motivation to players during difficult times.

Many of the reviewed games (eg, *Depression Quest* and *Before I Forget*) aimed to raise awareness and build empathy through immersive experiences that replicate the emotional realities of those affected. These games often use narrative-driven storytelling to allow players to live through the struggles of a character facing depression. Through experiencing the emotional highs and lows, players are encouraged to empathize with the character’s struggles. This emotional journey is argued to be crucial not only for players who are personally dealing with mental health challenges but also for their support networks—such as partners, family members, and colleagues—who may benefit from a better understanding of what their loved ones are going through. This approach can externalize internal emotional struggles, providing validation for players dealing with similar issues. The intention here is to not only entertain but offer a profound and empathetic experience that helps players feel less isolated.

Some reviewed games (eg, *MindMax* and *Stigma-Stop*) encouraged players to seek professional help for mental health concerns. By embedding choices in the game where players can seek therapy, use support resources, or talk to someone, these games guide players toward understanding the importance of professional help in managing mental health conditions.

Six games targeted adolescents and youth from 15 to 25 years, and one targeted younger children. Six games do not specifically target an age group, but board game players. Examples of these groups include 1 study for men who are interested in AFL games and 1 study for company managers.

Among these games, only 2 games targeted new mothers with a game intervention focused on promoting knowledge. Although few studies directly target new parents, there is arguably some overlap with the depressive symptoms faced by the other groups discussed above. However, new parents are often in a unique and vulnerable position, facing a combination of physical, emotional, and psychological challenges. They usually experience heightened vulnerability, exhaustion, and lack of time [67], which requires an intervention that is not only effective but also easily accessible and low-pressure. Many of the games targeting adults with depression do an excellent job of portraying the emotional complexities of mental health issues. Still, their intensity and focus on complex gameplay may not be suitable for new parents who are experiencing severe fatigue. It is argued that the new parent target audience would benefit more from short, accessible gameplay that can be easily integrated into their busy routines, and casual yet meaningful experiences that provide emotional validation and support.

Given the needs of the new parent target audience, mobile platforms are likely the most suitable choice. Mobile games can be played in short bursts, require minimal setup, and are highly accessible because most people carry smartphones. Mobile accessibility also allows parents to engage with the game whenever they find spare moments, which is crucial given their unpredictable and fatiguing schedules. However, a hybrid solution that combines mobile and web-based access to ensure that new parents have multiple ways to engage with the game based on their convenience may also be valuable. A web-based version could provide a larger screen and more detail for those moments when new parents can access a computer, while the mobile version allows instant play. WebGL technology further reduces minimum system requirements and will enable games to be accessed from any electronic device. Hybrid accessibility also increases reach, as it accommodates different user preferences and ensures that players have multiple ways to interact with the content.

Console and VR platforms, while highly immersive, are arguably not ideal for most new parents. While VR today is widely “plug and play” and often requires little more than a connection ID to get started, the transition to becoming a parent, particularly in the early months, is marked by extreme time constraints, chronic sleep deprivation, and a constant state of divided attention [68]. New parents often navigate caring for an infant, are adjusting to unpredictable routines, and manage both emotional and physical exhaustion. Therefore, it is proposed that even simple steps such as clearing space, troubleshooting hardware, or remaining engaged in a virtual environment for more than a few minutes can become overwhelming for parents who might be at risk of cognitive and emotional overload.

## Discussion

### Overview

There is a dearth of evidence directly related to PND, which is only 2 studies in the past 10 years. After expanding the search to depression, we included 11 more. The included studies predominantly aligned with 3 overarching themes: the promotion of knowledge, the reduction of stigma, and the raising of awareness. A core intention of several of the reviewed games (eg, *Above Water* and *Moving Stories*) is to educate players about mental health and reduce the associated stigma. This is achieved by exposing players to the symptoms, challenges, and lived experiences of individuals who are dealing with various mental health conditions. The games use different approaches, such as presenting players with scenarios that require them to make supportive decisions, simulating real-life interactions with people facing mental health challenges, and providing informative feedback. The ultimate goal is to educate the players on mental health literacy, that is, what different symptoms mean, why they matter, and how to effectively respond. This type of education, delivered in an interactive, engaging way, generally helped to demystify mental health issues, correct misconceptions, and encourage empathetic attitudes. This is argued to play a crucial role in reducing stigma by promoting understanding and highlighting the commonality of these experiences, breaking down the barriers that often prevent open conversations about mental health.

### Game Mechanics

For depression management in general, several mechanisms are argued to be particularly impactful. Narrative storytelling, as seen in games such as *Hellblade: Senua’s Sacrifice* and *Depression Quest*, may provide emotional engagement that helps players feel understood and validated by illustrating shared experiences of mental health struggles. Given the evidence that the recognition and acceptance of an individual’s feelings and experiences promote mental well-being, seeing their experiences reflected in the storyline may create an emotional connection that could combat isolation. Choice-based interaction, where players make decisions that affect the storyline, may offer a sense of agency and sense of control, which is often decreased in those experiencing depression [69]. These decisions can illustrate how positive actions, such as seeking help or practicing self-care, might lead to better outcomes, potentially reinforcing a sense of empowerment for new parents. Simulated real-life scenarios could also play a role in helping players practice practical coping skills, such as managing stress or communicating with loved ones in a safe and controlled environment. Combined with resilience mechanics that highlight small wins, these mechanics may help maintain motivation during challenging times. Additionally, low-pressure gameplay is likely important to this target group, as it may reduce the cognitive and emotional needs of interaction, making the game more accessible to new parents who are experiencing fatigue and time constraints.

For educational purposes and stigma reduction, specific game mechanics may help build knowledge and empathy in accessible ways and minimally overwhelm the player. For example, educational micromessaging, as seen in “Match Emoji” and “Stigma-Stop,” delivers brief and embedded content that increases awareness about mental health conditions. This mode of delivery may be especially valuable for new parents, who often benefit from concise, low-effort learning formats. Empathy-building mechanisms could also contribute to stigma reduction, an important function within PND given its high association with stigma [13]. Interactive elements that allow players to make supportive choices during character interactions may foster greater understanding and compassion. Role-playing as characters facing mental health challenges may help players consider these issues more personally, potentially encouraging self-compassion and empathy for others. Additionally, real-life scenario simulations may offer players opportunities to engage in realistic support-seeking situations, which could enhance a better understanding of seeking and receiving support.

Given the cultural, social, and personal barriers to seeking help for people with PND [13], certain game mechanics could play a valuable role in improving help-seeking behavior by reducing hesitancy and encouraging engagement with professional help. Choice-based interactions that include options for seeking therapy or speaking to a health care professional can illustrate the potential positive impact of these decisions and may help normalize the idea of reaching out. In-game scenarios that model conversations with health care professionals or trusted family members could also reduce anxiety by providing players with low-risk opportunities to observe or practice supportive dialogue. Additionally, incorporating positive reinforcement, such as visual or narrative cues that reflect improvements in well-being or relationships following help-seeking, may highlight the value of accessing help. For expectant parents who may experience PND after their baby is born, these mechanics may offer gentle encouragement and promote greater confidence in taking the first step. As shown in Table 6, here is a comparison of game mechanics and different aims.

**Table 6.** Comparison of game mechanics and different aims.

	Build knowledge	Build empathy	Build awareness	Reduce stigma
Narrative storytelling	✓	✓		✓
Choice-based interaction		✓		✓
Educational micromessage	✓		✓	
Character interaction	✓	✓		
Real-life scenarios	✓		✓	
Resilience mechanics			✓	

### Principal Findings

To effectively support new parents coping with PND and to address the gaps identified in this review, the authors argue that future research should focus on the interprofessional co-development of a serious game with the following important design elements. First, we argue that a relatable and engaging story is important and should reflect both the struggles and victories that new parents face, helping them see their own experiences mirrored in the game. By doing this, the game can foster a deep sense of understanding, connection, and emotional validation, which may be crucial for reducing the isolation [13] often felt during PND. Allowing players to make meaningful choices that influence the direction of the game could give them a sense of agency and control to combat the low sense of agency [69] frequently found in the experience of depression. These decisions should reflect real-life challenges, and the consequences should demonstrate how positive actions, such as seeking help or practicing self-care, can improve well-being. This interactive element can empower new parents to feel more in control of their own mental health journey. Incorporating real-life scenarios is also a key element. These scenarios should include common parenting challenges, such as communicating effectively with a partner, managing stress, or seeking support. Practicing these skills in a safe, controlled game environment can help players build confidence, making it easier to apply what they have learned to real life.

Gamification elements such as badges, progress tracking, and daily challenges should be used to keep players motivated and committed, in line with a positive reinforcement approach to learning and improving intrinsic motivation [70]. These features add an element of fun and reward, encouraging players to engage consistently with the game. The game should also include educational messages that provide important information about PND. These messages could cover recognizing symptoms, understanding when to seek help, and learning practical self-care techniques. Delivering these messages in small, easily digestible portions ensures that players can learn without feeling overwhelmed, and it is in line with research findings that show benefits for adult learners who engage in mobile-based microlearning steps [71].

Character interactions are another essential design element. Players should have opportunities to interact with supportive characters, which can help build empathy and promote positive communication skills. These interactions can model supportive relationships and reinforce behaviors that new parents can use in their own lives to improve their support networks. Celebrating small wins is also important; highlighting moments such as taking time for self-care or successfully reaching out for help can reinforce resilience and make players feel that each positive step, no matter how small, is a significant achievement. Lastly, the game should be designed to be accessible and easy to engage with in short sessions. Low-pressure gameplay is important, as new parents often face exhaustion and have limited time [68]. The game should be flexible, allowing players to pick it up and put it down whenever they have a few moments, ensuring it serves as a helpful, nonstressful tool in their daily lives. This balance of emotional engagement, practical learning, and ease of access is proposed to create a supportive experience that can make a meaningful difference for new parents coping with PND.

### Recommendations

#### Overview

Serving the aim of our proposed project, we recommend focusing on the following subset of game types and mechanics.

#### Narrative-Driven Storytelling

Incorporate an engaging storyline that reflects the real-life experiences of new parents, including both struggles and successes. This mechanic helps players feel understood and connected, reducing feelings of isolation. The narrative should also include resilience themes to promote hope and perseverance.

#### Choice-Based Interactions

Implement choices that directly influence the game’s storyline, allowing players to make decisions that affect their mental health journey. This empowers players by giving them a sense of control, emphasizing the positive outcomes of seeking help or practicing self-care.

#### Real-Life Scenario Simulations

Include scenarios that simulate everyday parenting challenges, such as communicating with a partner, managing stress, or seeking professional support. Practicing these skills in a game setting builds players’ confidence and provides them with practical tools for real-life application.

#### Low-Pressure, Casual Gameplay

Design the game for short, manageable sessions that can be played on a mobile device. This mechanic ensures accessibility for new parents, allowing them to engage with the game without added pressure or stress. Casual mechanics make the game easier to pick up and play whenever parents have a few free moments.

#### Educational Micromessaging

Integrate educational content subtly throughout the game to provide important information about PND, such as recognizing symptoms and self-care tips. Delivering these messages in a digestible format helps fill knowledge gaps without overwhelming the player.

#### Character Interactions and Empathy-Building

Allow players to interact with characters representing supportive individuals, such as partners, family members, or health care professionals. These interactions help model healthy communication and build empathy, both for oneself and others.

#### Gamification Elements

Add elements such as badges, progress tracking, and daily challenges to encourage ongoing engagement. Gamification makes the experience more rewarding and helps players stay motivated, which is especially important for building positive habits.

#### Celebrating Small Wins

Highlight small victories in the game, such as successfully reaching out for support or completing a relaxation exercise. Positive reinforcement builds resilience and emphasizes that progress, no matter how small, is meaningful.

### Strengths and Limitations

This review draws strength from its interdisciplinary synthesis of literature spanning digital mental health interventions and serious games, offering a comprehensive overview that bridges technological and psychological perspectives. Examining a novel and emerging approach to PND care contributes to a growing area of interest that remains underexplored in current research. In instances where PND-specific evidence is limited, the review draws from broader depression literature to extract relevant insights while maintaining awareness of contextual differences. Importantly, it also provides a forward-looking perspective by identifying clear gaps in the existing literature and outlining specific directions for future research. These include the development and tailoring of serious games for PND populations, informed by existing evidence on effective game mechanics in comparable mental health contexts.

However, this review also has several limitations. First, the limited number of studies specifically focused on PND constrained the depth of analysis and made it necessary to draw from broader depression literature. While this allowed for the extraction of relevant insights, it may affect the generalizability of findings to the unique psychological and social experience of individuals with PND. Second, there is a potential for selection bias in the inclusion of literature; however, attempts to reduce this included using a structured search process and adhering to the preagreed scope. Future reviews might consider incorporating elements of systematic review methodology, such as dual screening or formal bias appraisal, to enhance rigor. Third, the review was limited to English-language publications, which may have excluded relevant studies from culturally and linguistically diverse populations, potentially narrowing the applicability of findings across global contexts. Expanding the language scope in future reviews could provide a more inclusive understanding of how serious games may support help-seeking across varied cultural settings. Finally, while a narrative review design can lack a systematic or quantitative appraisal of study quality and findings, in this case, given the heterogeneity of the studies reviewed, it did allow for a comprehensive synthesis of the available literature to better pinpoint specific targeted areas needing additional research with recommendations for targeted and novel game design to address that identified gap.

### Future Directions

Based on the findings of this review, the authors plan to develop a game design prototype that selectively incorporates these recommended game mechanics, which will allow for an empirical investigation of utility and efficacy. The game design will have a particular focus on the accessibility and usability needs of new parents. We will begin by conducting user research, including interviews with new parents, to further understand their challenges and preferences. This process will ensure that the game design aligns well with their experiences and provides meaningful support. We will also collaborate interprofessionally with mental health professionals to ensure that the educational content is accurate and that the overall gameplay promotes mental well-being.

Additionally, we aim to develop a hybrid solution that allows for both mobile and web-based accessibility, ensuring that new parents have multiple ways to engage with the game based on their convenience. Once the prototype is ready, we will conduct usability testing and content value testing to assess its possibility and feasibility in engaging new parents and providing mental health support. Feedback from this testing phase will guide further refinements to make the game as supportive and accessible as possible for new parents to improve help-seeking behavior for PND.

### Conclusions

The evidence base for PND-related interventions is currently limited and emerging, highlighting a notable gap in future research. Based on the analysis of existing games, many of them aim to educate players on mental health topics, using engaging scenarios that require players to make supportive decisions and learn about the lived experiences of people with mental health issues. This approach encourages players to understand mental health, corrects misconceptions, and promotes empathy, ultimately reducing stigma. Narrative storytelling also plays a significant role, allowing players to experience the emotional highs and lows of a character’s journey, fostering a deep connection and validation for players facing similar struggles. Empowering players was also found to be valuable in seeking professional help by embedding scenarios that encourage reaching out to health care professionals and practicing coping skills in a low-risk, game-based environment. It is argued that these mechanics help players feel more comfortable about seeking support and build their confidence in managing their own mental health.

The included games target a range of audiences, including adolescents, adults, and the general public, identifying a literature gap that only a small number are specifically designed for new parents. New parents face unique challenges, such as exhaustion and time constraints, suggesting the ideal game platform is likely to be mobile—rather than immersive—platforms as they offer the flexibility and ease of use necessary for new parents. Finally, this review underscores the importance of expanding serious game research to more effectively reach and support those experiencing PND, a population that remains inadequately treated and frequently stigmatized in modern culture.

## Supplementary material

10.2196/70777 Multimedia Appendix 1 Detailed search strategies.

10.2196/70777 Checklist 1 PRISMA-ScR checklist.

## Abbreviations

CBT: cognitive behavioral therapy
CVG: casual video game
*DSM-5*: *Diagnostic and Statistical Manual of Mental Disorders*, Fifth Edition
mHealth: mobile health
PND: postnatal depression
VR: virtual reality
