# Supplementary material for: Exploring Serious Games in Supporting Postnatal Depression: Narrative Review
Source: Interact J Med Res. 2026 Jan 16;15:e70777. doi: 10.2196/70777 (PMC12810747; doi:10.2196/70777)
Supplement: Multimedia Appendix 1 — Detailed search strategies. [file ijmr-v15-e70777-s001.docx]

| Set | Search Statement |
| --- | --- |
| 1. | Depression, Postpartum/ |
| 2. | Depressive Disorder/ or Depression, Postpartum/ or Pregnancy Complications/ or Perinatal Care/ or Depression/ |
| 3. | Depression/ or Depression, Postpartum/ or Mothers/ or Humans/ or Depressive Disorder/ or Female/ or Adult/ |
| 4. | Depressive Disorder/ or Depression, Postpartum/ or Depression/ |
| 5. | 1 or 2 or 3 or 4 |
| 6. | User-Computer Interface/ or Learning/ or Video Games/ or Computer-Assisted Instruction/ |
| 7. | Games, Experimental/ or Gamification/ or Video Games/ or Mobile Applications/ |
| 8. | game design.mp. |
| 9. | Learning/ or Games, Experimental/ or Video Games/ or Adult/ |
| 10. | Video Games/ or Internet/ |
| 11. | 6 or 7 or 8 or 9 or 10 |
| 12. | 5 and 11 |
